# The Book World of Medicine and Science

**Published:** 1903-05-09

**Authors:** 


### The Book World of Medicine and Science.

The Diagnosis and Modern Treatment of Pulmonary Consumption, with Special Reference to the Early Recognition and the Permanent Arrest of the Disease. By Arthur Latham, M.A., M.D.Oxon., M.A.Cantab., Assistant Physician at the Brompton Hospital for Consumption and Diseases of the Chest, etc. (Pp. 215. Demy 8vo. London: Baillière, Tindall and Cox. 1903. Price 5s. net.)

In this excellently printed and generally attractive volume Dr. Arthur Latham has collected and conveniently arranged a series of articles published in various journals during the last few years. They appear at an opportune time, and although devoid of any conspicuous originality, and in parts somewhat marred by a too dogmatic manner of presentation, nevertheless afford a useful epitome of much of the best views regarding the most reliable methods for the early recognition, and the most rational and effective procedures for securing the arrest, of pulmonary tuberculosis. The pathological aspects of the subject are unfortunately all too inadequately expressed, and, indeed, the value of the work throughout would have been much enhanced by a more careful presentation of the most important pathological facts, an intimate knowledge of which must necessarily form the basis for further advance not only in prophylaxis but also in diagnosis and treatment. In the section dealing with the diagnosis of the chronic varieties, the fibro-caseous or ordinary, the fibroid and irregular forms are described. A special chapter is devoted to the very important matter of securing satisfactory procedures for the avoidance of reinfection. Dr. Arthur Latham, while allowing that Koch has shown that human tubercle bacilli are less virulent than those derived from bovine sources, contends that we should still consider that tubercle bacilli derived from cow's milk and other animal sources may convey infection to man. The measures to be adopted in dealing with the tubercle bacilli are indicated, but no royal road is pointed out as to the manner in which wayward man is to be compelled to practise hygienic precept. At the present time the most interesting portion of the volume is that dealing with the principles of the open-air method of treatment as carried out in a sanatorium. Dr. Latham has evidently taken much pains to acquaint himself with the theoretical considerations presented by his subject, but we venture to think that resident medical officers connected with such institutions will be impressed with the fact that the author views the subject rather from the side of the critical outside observer than the internal and essentially practical one of a medical director, in intimate contact with the practical life and oftentimes perplexing working, and very real worries of a sanatorium. Those who have studied the psychology of the phthisical in institutions will not, we imagine, altogether agree with Dr. Latham that "the more restricted the accommodation for visitors the better, as their absence seldom leads to harmful results, whilst their presence is often injurious," and some will think he is rather too strong in his condemnation of certain amusements. We are glad to find the value of judicious hydro-therapeutic measures acknowledged although only the most meagre descriptions are given. As regards the requirements necessary in the building, equipment, and practical management of a sanatorium, Dr. Latham evidently writes merely as a well-informed and intelligent onlooker. Many of his much too dogmatically expressed opinions will be opposed by those who work, as it were, from the inside. Dr. Latham, one would imagine, cannot have made very extensive inquiries from those having the management of young people in our public institutions for early cases of phthisis, or he would not say "there is no necessity for separating the men and women in the dining-room or grounds." Many useful suggestions will be found in the section dealing with the details of the open-air method of treatment as carried out in a private house. Interesting examples of menus for the chief meal of the day at existing sanatoria are given, but we cannot understand why Christmas Day should have been selected in one instance as it could hardly be other than an "extraordinary" example. Many of the other forms of treatment employed during recent years receive brief and not always altogether adequate consideration. The use of urea is mentioned, but the results of the careful observations of Dr. Vere Pearson and others, which go far to demonstrate the uselessness of this agent, are not referred to. The chapter on the treatment of special symptoms contains many suggestions likely to be of service to practitioners. As Dr. Latham shows, drugs are but rarely needed for the control of profuse perspiration in a well-conducted sanatorium, for, with the adoption of open-air methods and an abundant food supply, night sweats almost invariably disappear. Concerning the very difficult and perplexing matter of the indications which should guide us in the selection of cases suitable for treatment in public and private sanatoria as at present available, Dr. Latham is all too brief. We are also inclined to take exception to his advice that "as soon as the disease has become arrested, tubercle bacilli have disappeared from the sputum, and constant medical supervision is no longer required, consumptive individuals may be advised to marry provided that they are able to live under suitable conditions." The value of the work as a permanent source of reliable information is much lessened by the absence of references to recent literature concerning the matters discussed. But in spite of numerous drawbacks and manifest limitations the work is one deserving of careful study, and is likely to do much in encouraging a rational consideration of the hygienic treatment of pulmonary consumption.

Rate-supported v. Voluntary Hospitals: A Comparison between Rate-supported and Voluntary Systems, on which is based an Argument for the Increased Support of Voluntary Hospitals. By J. G. Craggs, M.V.O., F.C.A. (London: The Scientific Press, Limited. 1s. 6d. net.)

This is a pamphlet of 37 pages which deals with hospitals supported by the rates, the Metropolitan Asylums Board system, small-pox statistics, a summary of the reports and expenditure of the Asylums Board, the London infirmaries, voluntary hospitals, the inequity of the subscription list, a general hospital rate and its probable amount, why limited liability companies should contribute to hospitals, and the special claims of the latter on life insurance companies, joint-stock companies, and railway companies. It is well printed, eminently readable, and contains much matter well worth weighing by experts and the public generally.

